# Tobacco

**Published:** 1874-04

**Authors:** J. W. Mason

**Affiliations:** Detroit.


### SELECTED ARTICLES.

---

### ARTICLE IV.

### *Tobacco.*

Read before the Detroit Academy of Medicine.

BY J. W. MASON, M. D., DETROIT.

Tobacco was discovered by Columbus and his crew, at Cuba, in 1492, and was introduced by him into the old world, and the "lightning speed with which its use spread over the earth is one of the greatest miracles in the history of commerce, and the coincident appetite of the human family." And, notwithstanding the powerful opposition that it has received from reformers, and even royal edicts, it has maintained itself against their wrath, and, to-day, the fascinating fumes of the weed hold in abject slavery millions of human beings.

In the composition of tobacco the most essential element, or principle, is nicotine. It is a colorless, liquid alkaloid, and has a burning, acid taste. It is one of the most intense of all poisons, approaching in its activity to hydrocyanic acid. The next most important element is a concrete volatile oil, called nicotianin, which is also an intense, active poison, differing, however, essentially from the alkaloid, and is supposed to act differently on the vital organs.

It is said that chewers get less of the oil but more of the alkaloid, while the smoker gets more of the oil, and therefore claims that as he does not get the deadly alkaloid, the practice of smoking is less detrimental than that of chewing. But Prof. Morries has shown, in a series of analytical examinations, that tobacco smoke does contain nicotine, and that, if it were allowed to accumulate in the blood, the act of smoking would soon be fatal. Headland says: "It is certainly absorbed to some extent, but it passes quickly into the urine, where it may be detected by a simple chemical test." We are aware, however, that the introduction of but a very small quantity in the system will produce marked indications of intoxication in those who are not accustomed to its use. And that it is only after a morbid condition of the system has been produced, that fragrant fumes can be inhaled with any degree of safety or satisfaction.

In habitual smokers—those who have become "iron clad" —it often excites thirst and an increased flow of saliva; but being an exhilarant, it soon produces, says Pereira, "a remarkable soothing and tranquilizing effect on the mind, which has made it so much admired and adopted by all classes of society, and by all nations, civilized and barbarous." But this tranquilizing effect, says Headland, is nothing more or less than "inebriation or drunkenness, which may exist in various degrees."

There is no need of much physiological acuteness to account for the bad effects that this pernicious habit has on the health of the constant consumer, when we take into consideration that tobacco is a powerful narcotic poison, which when brought in contact with an absorbing surface like the mucous membrane, cannot fail to arrest or retard all the more minute molecular changes which are constantly taking place in the healthy state of the living tissue.

I am unable to determine to what extent the practice of chewing conduces to the production of disease, but there is any amount of medical evidence showing the baneful effects of smoking. Dr. Prout says that it "disorders the assimilating functions in general, and particularly the assimilation of the saccharine principle." Monsieur Fiévée, an eminent French physiologist, claims that the use of tobacco smoke develops numerous constitutional diseases, among which he enumerates dyspepsia, congestion of the brain, apoplexia, palsy, mania-a-potu, insanity, nervousness, amaurosis, and emasculation. He also adds, there exists "a danger of far greater interest to those concerned in the preservation of the individual, in the enfeeblement of the human mind, the loss of the powers of intelligence and of moral energy: in a word, of the vigor of the intellect, one of the elements of which is memory."

An eminent London physician says that "smoking tobacco weakens the nervous powers, favors a dreamy, imaginative, and imbecile state of existence, and sinks its votary into a state of careless or maudlin inactivity, and selfish enjoyment of his voice." Prof. Laycock corroborates these observations, and says that the "inveterate habit of smoking is worthy the special notice of physicians, as a very frequent but unconsidered and unthought of cause of disease."

In my own professional experience, I can recall to mind many cases where the pathological conditions were undoubtedly induced by excessive smoking; and I could present hundreds of cases, reported by eminent physicians, where disease, and even death, has been produced by the use of tobacco.

Trousseau and Bretonneau, both eminent French physicians, have reported many fatal cases, which they attribute to the use of tobacco. Berat, the French poet, was one of the cases.

The late Dr. Zina Pitaher, in 1855, reported a case of delirium tremens occasioned by the excessive use of tobacco. Dr. Bucknill, in 1843, reported eight cases of insanity received into the Massachusetts State Hospital, produced by the use of tobacco. Dr. Kirkbride, of the Pennsylvania Hospital for the Insane, also reported four cases of insanity in 1849, caused by the use of tobacco. The use of tobacco, he says, "has, in many individuals, a most striking effect on the nervous system, and its general use in the community is productive of more serious effects than is commonly supposed."

Results of this kind are quite in keeping with what we know of the physiological properties of the oil and alkaloid of tobacco. It is reasonable to expect that the practice of volatilizing tobacco, so powerful a sedative poison, and applying its vapor to the lining membrane of the air passages, would produce derangement of the nervous system, varying in intensity according to the great or less dilution with atmospheric air.

King James, in his celebrated counterblast against the use of tobacco, says "that smoking is a custom loathsome to the eyes, baneful to the nose, harmful to the brain, dangerous to the lungs, and in the black, reeking fumes thereof, nearest resembling the horrible Stygian smoke of the pit that is bottomless."

Having brought forward evidence enough to convince the most skeptical that the use of tobacco is not only a foolish, but a most deleterious habit, I will only present one individual case of the somatic effects of tobacco for your consideration.

Mr. G., a young man of twenty-six, who was an inveterate smoker, and in whose constitution the nervous and lymphatic temperament are singularly blended, came under my observation June 23, 1873. Without any promonitory indications, he was suddenly attacked with spasms, attended with severe cramps of the extremities, nausea and vomiting. The spasms lasted from five to ten minutes, during which time he was apparently unconscious. As soon as the paroxysm passed off he would regain his consciousness but there was a remarkable depression of spirits, with great prostration of muscular strength. The remission lasted from fifteen to twenty minutes.

After several seizures, the pulse became weak and frequent, the skin cool and bedewed with sweat; the countenance was pallid, and eyes sunken in the sockets. The feet and legs became ice cold, and at one time I had grave apprehensions for the safety of my patient, but, after nearly two hours of active application of dry heat, and gentle friction to the extremities, aided by a hypodermic injection of morphia, and a large sinapism over the stomach and bowels, with the administration of small doses of brandy, tinct. ginger, and aqua menth. pip., I had the satisfaction of seeing a radical improvement in my patient.

After he was relieved of the malady, the skin soon regained its natural temperature, the haggard expression of countenance passed away, and he rallied rapidly from the nervous and physical prostration.

In the morning, after a refreshing night's sleep, he arose feeling nearly as well as ever. He partook of a moderate breakfast, and then resumed his pipe; but, unfortunately for him, the fragrant fumes did not produce the tranquilizing and soothing effect spoken of by Pereira, but produced a recurrence of the malady that he had suffered so fearfully with the day before.

Believing that smoking was the proximate cause of his difficulty, I strictly enjoined on him abstinence from the further use of it until, at least, he had regained his wonted vigor. But about four P. M., he was again tempted by the overwhelming power that the fascinating fumes of the weed had over him, to resort to its use again. The result was the same as before, only more severe. He remained for a long time unconscious, after the spasms passed off. The coldness and muscular prostration was even greater than in the first instance, and it was with great difficulty that reaction was effected.

After reaction took place he convalesced rapidly, and, having forsworn his pipe, was not troubled by any further recurrence of the malady.—*Detroit Review of Medicine and Pharmacy.*

